# Softness, Elasticity, and Toughness of Polymer Networks with Slide-Ring Cross-Links

**DOI:** 10.3390/gels7030091

**Published:** 2021-07-13

**Authors:** Koichi Mayumi, Chang Liu, Yusuke Yasuda, Kohzo Ito

**Affiliations:** 1 The Institute for Solid State Physics, The University of Tokyo, 5-1-5 Kashiwanoha, Kashiwa, Chiba 277-8581, Japan; changliu@molle.k.u-tokyo.ac.jp; 2 Material Innovation Research Center (MIRC), Department of Advanced Materials Science, Graduate School of Frontier Sciences, The University of Tokyo, 5-1-5 Kashiwanoha, Kashiwa, Chiba 277-8561, Japan; y.yasuda@molle.k.u-tokyo.ac.jp

**Keywords:** mechanical property, fracture toughness, polyrotaxane, slide-ring gel

## Abstract

Slide-ring (SR) gels cross-linked by ring molecules are characterized by softness (low Young’s modulus), elasticity (low hysteresis loss), and toughness (large fracture energy). In this article, the mechanical and fracture properties of SR gels are reviewed to clarify the physical understanding of the relationship between the molecular-level sliding dynamics of the slide-ring cross-links and macroscopic properties of SR gels. The low Young’s modulus and large fracture energy of SR gels are expressed by simple equations as a function of the degree of sliding movement. The dynamic fracture behaviors of SR gels gives us the time scale of the sliding dynamics of the cross-links, which is at the micro-sec scale. The fast sliding motion of the cross-links leads to the elasticity of the SR gels. The SR concept can be applied to solvent-free elastomers and composite materials.

## 1. Introduction

The cross-linking of polymers is an important process for improving the mechanical elasticity, toughness, and stability of polymeric elastomers. Conventional cross-linking methods are classified into two categories: chemical and physical cross-linking. Chemical cross-linking is formed by covalent bonds between polymer chains. The covalent cross-links are stable under deformation, which leads to high stiffness (high Young’s modulus) and mechanical elasticity of chemically cross-linked elastomers [1]. However, as the chemical cross-linking density increases, the partial chain length between cross-links becomes shorter and the mechanical toughness is reduced according to Lake Thomas theory [2,3]. In contrast, physical cross-links are non-covalent bonds, such as hydrogen bonds, ionic interactions, coordinate bonds, and dynamic covalent bonds [1,4,5,6,7,8,9,10,11,12,13]. Physical cross-links are weaker than the covalent counterparts and are broken under deformation. Breakage of the physical cross-links dissipates strain energy, similar to sacrificial bonds in double-network gels [14,15], and the energy dissipation can improve the mechanical toughness of the elastomers [16,17]. However, the dynamic rupture of the physical cross-links results in residual strains and mechanical hysteresis under repeated cyclic deformation [16,17]. Realizing both high elasticity (low mechanical hysteresis) and toughness is a challenging issue for the material design of polymeric elastomers.

**Figure 1:**
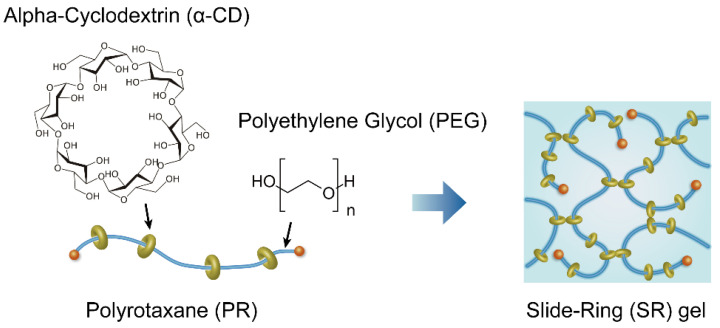
Schematic illustrations of PR and SR gels. Reprinted from [31] with permission from Elsevier.

A novel type of cross-linked polymer, a slide-ring (SR) material, was developed by Ito et al. [18]. Okumura and Ito fabricated SR gels by cross-linking ring molecules of polyrotaxanes (PRs), in which ring molecules are threaded onto an axial polymer chain (Figure 1) [19]. In SR gels, the axial polymer chains are cross-linked by figure-of-eight cross-links, and the cross-linking points can slide along the polymer chains. Interestingly, SR gels exhibit low Young’s modulus [20,21,22,23,24,25,26,27], high toughness [20,23,28,29,30,31,32], and elastic mechanical response (low hysteresis) [20,23,28,33], overcoming the trade-off between toughness and elasticity for conventional polymeric elastomers.

Herein, we review the mechanical and fracture properties of SR gels to clarify the correlation of the sliding of the movable cross-links with the softness, elasticity, and toughness. We first explain the preparation procedure of SR gels (Section 2). In Section 3, experimental and simulation results on the softness of SR gels are shown, and a simple molecular theory for the low Young’s modulus of SR gels is introduced. In Section 4, we focus on the quasi-static fracture behaviors of SR gels and give a simple fracture model to connect the high fracture energy with the sliding distance of the cross-links. The time scale of the sliding dynamics of the cross-linking points is discussed based on the dynamic fracture behaviors of SR gels (Section 5), which is related to the elastic mechanical responses of SR gels (Section 6). In Section 7, we introduce some examples of solvent-free SR elastomers.

## 2. Preparation of SR Gels

A typical example of PR for SR gels is composed of polyethylene glycol (PEG) and α-cyclodextrin (CD) (Figure 1) [18,19]. By cross-linking hydroxyl groups of different CDs via bifunctional cross-linkers, the PEG main chains are connected by figure-of-eight cross-links formed by two CDs. The sliding of the cross-links is influenced by two parameters of PR, PEG chain length [23] and CD coverage on PEG chains [28]. In this review, we focus mainly on SR gels prepared from PR with 25% CD coverage and PEG molecular weight of 35,000 g/mol [23,29,30,31]. The PR was dissolved in dimethyl sulfone (DMSO) with the PR concentration of 10 wt%, and hexamethylene diisocyanate (HDMI) was added to the PR solution as a cross-linker [23,29,30,31]. The cross-linking density of the SR gels was tuned by changing the cross-linker concentration. As a control, fixed cross-link (FC) gels were prepared by cross-linking pullulan in the same procedure as that of SR gels [23,29,30,31]. In Section 3, the PEG chain length dependence of the fracture toughness will be discussed based on the experimental results of SR gels with different PEG molecular weights from 35,000 g/mol to 100,000 g/mol [23]. SR hydro gels are prepared by cross-linking PRs with divinyl sulfone (DVS) in NaOH aqueous solutions [28]. The fracture properties of SR hydro gels with 25% and 2% CD coverages will be shown in Section 3.

## 3. Softness of SR Gels

**Figure 2:**
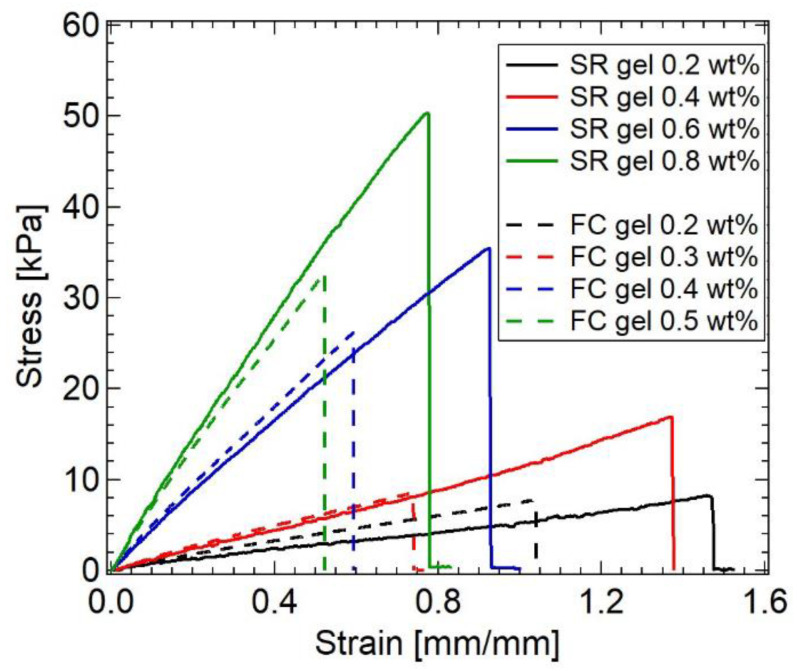
Stress–strain curves for FC and SR gels with different cross-linker concentrations. Reprinted from [23] with permission from American Chemical Society.

**Figure 3:**
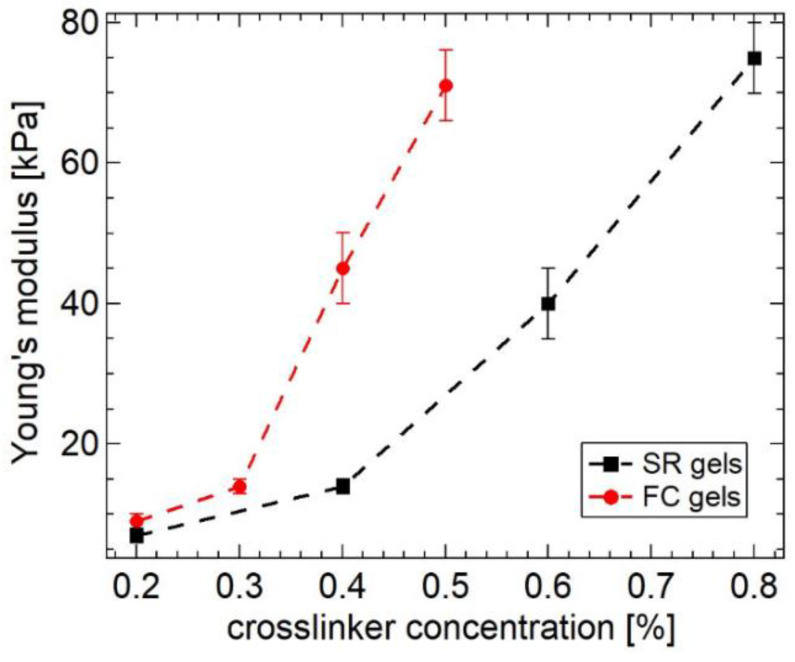
Cross-linker concentration dependence of Young’s modulus for FC and SR gels. Reprinted from [23] with permission from American Chemical Society.

The slidability of the cross-linking points leads to a softer mechanical response of SR gels compared with that of FC gels with fixed cross-links [20,21,22,23,24,25,26,27]. Figure 2 shows stress–strain curves of FC and SR gels with various cross-linker concentrations [23]. From the initial slopes of the stress–strain curves, the Young’s moduli of the FC and SR gels were estimated and plotted against cross-linker concentration (Figure 3). The Young’s moduli of the SR gels exhibit lower Young’s moduli than those of the FC gels, suggesting the softer mechanical response of SR gels.

**Figure 4:**
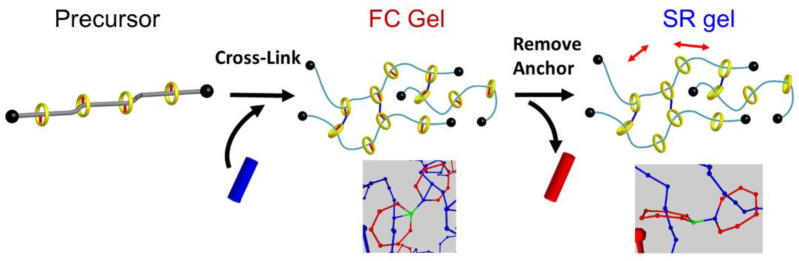
Schematic illustration of coarse–grained models for FC and SR gels. Reprinted from [27] with permission from American Chemical Society.

**Figure 5:**
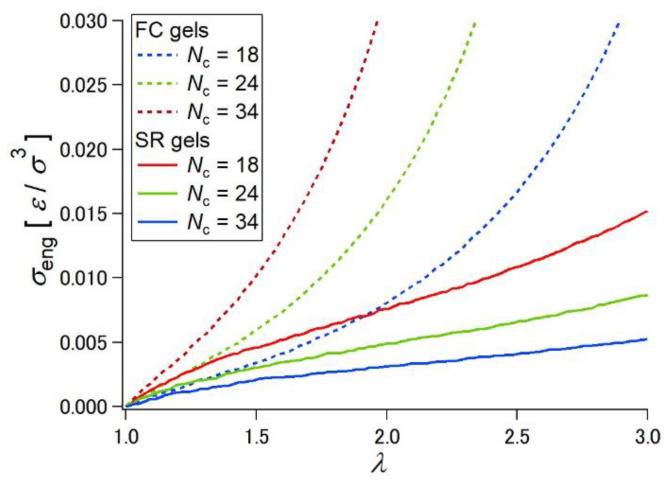
Stress–extension ratio curves obtained from MD simulations for SR and FC gels with different cross-linking densities. Reprinted from [27] with permission from American Chemical Society.

**Figure 6:**
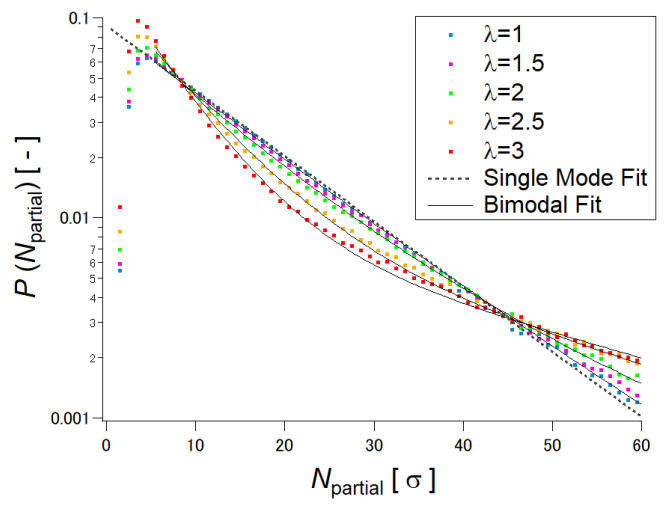
Distribution of segment number between cross-links for SR gel under uniaxial deformation. Reprinted from [27] with permission from American Chemical Society.

**Figure 7:**
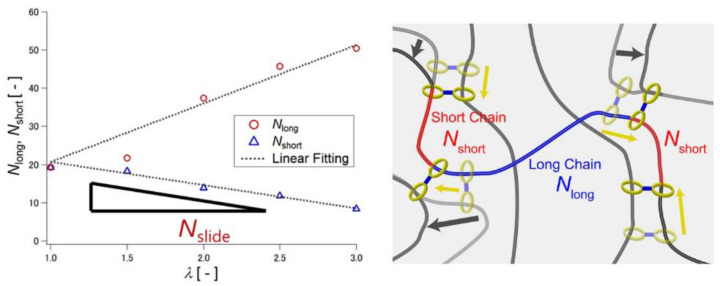
Relationship between extension ratio *λ* and segment numbers of shorter partial strands (*N*_short_) and longer partial strands (*N*_long_) under uniaxial deformation of SR gel. Reprinted from [27] with permission from American Chemical Society.

**Figure 8:**
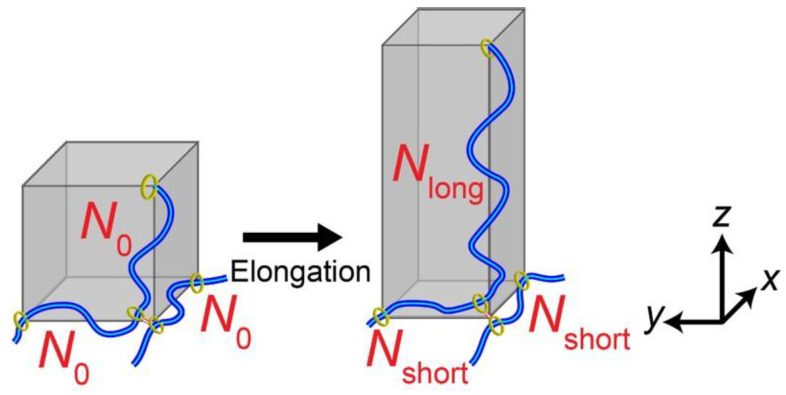
Schematic illustration of 3-chain model for SR gels. Reprinted from [27] with permission from American Chemical Society.

In order to understand the relation between the sliding motion of the cross-links and macroscopic mechanical properties of SR gels, Yasuda et al. performed molecular dynamics (MD) simulations of SR gels [27]. They developed a coarse-grained model of the SR networks based on the Kremer–Grest bead-spring model. As shown in Figure 4, FC gels were converted into SR gels by changing the fixed cross-links to figure-of-eight cross-links in order to compare FC and SR gels with the same cross-linking densities and structures. Figure 5 shows the simulated stress (*σ*)–extension ratio (*λ*) curves of SR and FC gels with various cross-linking densities. The SR gels exhibited lower stress than the FC gels. In order to quantify the sliding behavior of the cross-links in the SR gels under stretching, the distribution of the strand length (segment number) between the slidable cross-links, *N*_partial_, was evaluated. With increasing extension degree, the distribution of *N*_partial_ changed from a single exponential random distribution to bimodal distributions (Figure 6), which suggests that the sliding of the cross-linking points under uniaxial deformation leads to splitting of the network strands into shorter and longer chains. The chain lengths (segment numbers) of shorter and longer strands, *N*_short_ and *N*_long_, change linearly with the extension ratio *λ* (Figure 7). The slope of *N*_partial_ against *λ* is defined as *N*_slide_, corresponding to the degree of sliding in the SR gels.

(1) $$N_{\mathrm{short}}\left(\lambda \right)=N_{0}-N_{\mathrm{slide}}\left(\lambda -1\right)$$

where *N*_0_ is the average segment number of network strands in the undeformed state. Based on the results, a 3-chain model with free junctions was proposed for the SR gels (Figure 8) [27]. The ring junctions allow the chain segments to move from the *x*- and *y*-directions to the *z*-direction (stretching direction) under uniaxial deformation. The segment numbers of strands, *N_i_* (*i* = *x*, *y*, *z*), changes

(2) $$N_{x}\left(\lambda \right)=N_{y}\left(\lambda \right)=N_{0}-N_{\mathrm{slide}}\left(\lambda -1\right)$$

(3) $$N_{z}\left(\lambda \right)=N_{0}+2N_{\mathrm{slide}}\left(\lambda -1\right)$$

Movement of the chain segments through the free junctions weakens the chain extension in the stretching direction, and the deformation of the network structure becomes more isotropic under uniaxial stretching, as observed experimentally by small-angle X-ray scattering [20,34]. This leads to a lower Young’s modulus of SR gels than that of conventional chemical gels [23,24]. Assuming a Gaussian chain conformation and affine deformation, the Young’s modulus of the SR network, *E*_SR_, is given by a simple equation [27]:

(4) $$E_{\mathrm{SR}}=E_{\mathrm{affine}}{\left(1-\frac{N_{\mathrm{slide}}}{N_{0}}\right)}^{2}$$

**Figure 9:**
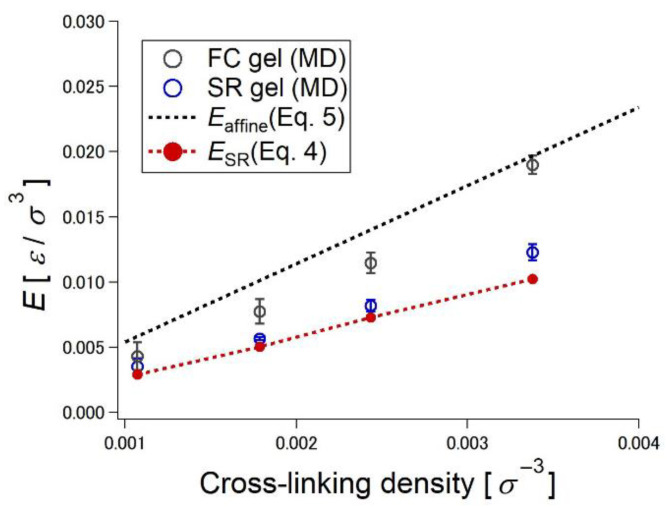
Cross-linking density dependence of Young’s modulus estimated by Affine network model (Equation (5)), three-chain model for SR networks (Equation (4)), and MD simulations of FC and SR gels. Reprinted from [27] with permission from American Chemical Society.

*E*_affine_ is the Young’s modulus from classical rubber elasticity theory for the affine network model:

(5) $$E_{\mathrm{affine}}=3\upsilon k_{B}T$$

where *ν* is the number density of network strands. According to Equation (4), the degree of sliding, *N*_slide_, is the dominant contributor to the deviation of the Young’s modulus from the value predicted by the affine network theory. Figure 9 shows the cross-linking density dependence of the Young’s moduli calculated from the theoretical predictions (Equations (4) and (5)) and the simulated stress–extension ratio curves in Figure 5. The Young’s moduli of the FC gels are close to the theoretical line of the affine network model (Equation (5)). In contrast, the Young’s moduli of the SR gels are below those of the FC gels, and the simulated Young’s moduli of the SR gels are almost the same as those calculated from Equation (4) by using *N*_slide_ obtained from the MD simulation. The simple equation, Equation (4), describes the relationship between the sliding of the cross-links and macroscopic mechanical properties of the SR gels.

## 4. Toughness of SR Gels

**Figure 10:**
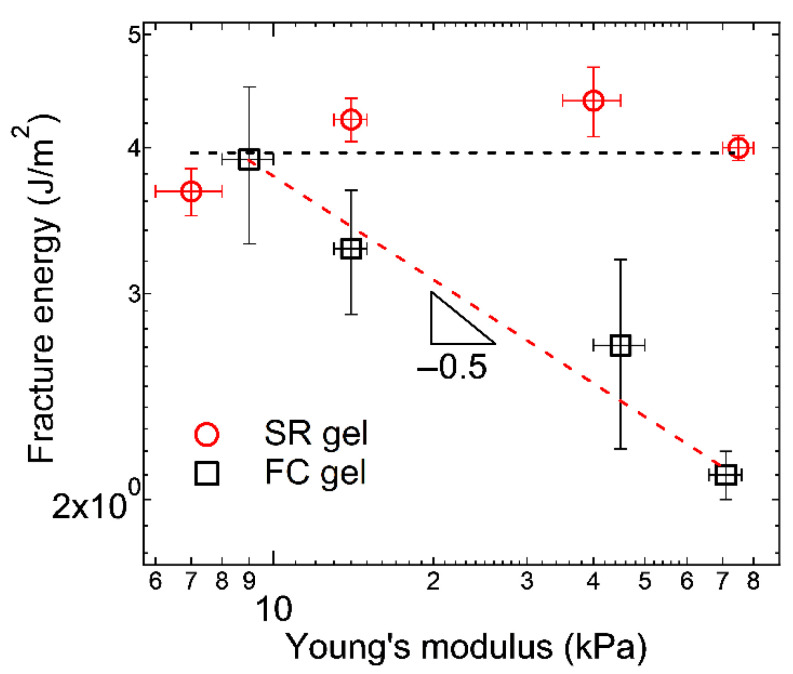
Young’s modulus dependence of fracture energy for FC and SR gels. Reprinted from [23] with permission from American Chemical Society.

**Figure 11:**
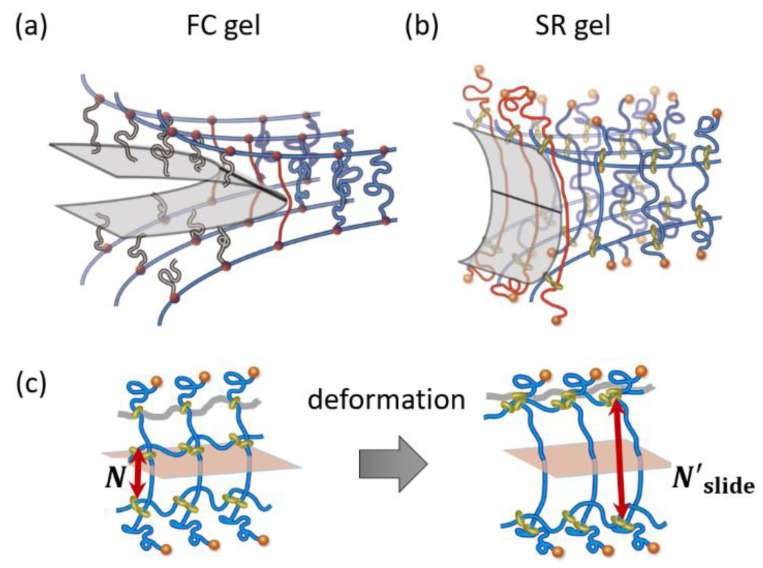
Schematic illustrations of crack propagation in (**a**) FC and (**b**) SR gels, and (**c**) transformation of strand length by sliding of cross-links in SR gels. Reprinted from [23] with permission from American Chemical Society.

The sliding of the cross-linking points also enhances the extensibility and toughness of polymer networks. The tensile stress–strain curves in Figure 2 indicate that the SR gels exhibit higher stress and strain at rupture than the FC gels, suggesting the higher mechanical toughness of the SR gels. For a more quantitative comparison of the toughness, Liu et al. carried out quasi-static fracture measurements of single-edge notch specimens of the SR and FC gels with various Young’s moduli (cross-linking densities). As shown in Figure 10, the measured fracture energies of the SR gels are larger than those of the FC gel. When a single-edge notched specimen of a FC gel is stretched, the polymer strand at the crack tip is fully stretched and then broken, which results in crack propagation (Figure 11a). According to the classic Lake–Thomas theory [3], the fracture energy of FC gels *Γ*_FC_ is given by the following equation:

(6) $$\Gamma_{\mathrm{FC}}={\left(\frac{3}{8}\right)}^{1/2}\nu L\;NU={\left(\frac{3}{8}\right)}^{1/2}n\;NU$$

where *ν* is the number density of network strands between neighboring cross-links, *L* is the end-to-end distance of one network strand, *N* is the monomer number of one network strand, *U* is the energy required to rupture one monomer, and *n* is the number of network strands passing through the unit area of a pre-crack surface, which is given by *νL*. By combining *L*∝*N*^−0.5^ for Gaussian chains and *N*∝*ν*^−1^∝*E*^−1^ for classical rubber elasticity theories with Equation (6), the relationship between the fracture energy *Γ*_FC_ and Young’s modulus *E* is given as follows:

(7) $$\Gamma_{\mathrm{FC}}\propto \nu^{-1/2}\propto E^{-1/2}$$

which is consistent with the estimated *Γ* of the FC gels (Figure 10). For FC gels, increasing the cross-linking density (i.e., Young’s modulus) reduces the toughness owing to the decrease of network strand length between cross-links.

For SR gels, the strand length at the crack tip is enlarged by sliding of the cross-linking points (Figure 11b,c). The increase in the strand length enhances the crack resistance and fracture energy. The fracture energy of SR gels, *Γ*_SR_, is expressed by the following equation:

(8) $$\Gamma_{\mathrm{SR}}=n\;{N^{\prime }}_{\mathrm{slide}}U$$

Here, *N*′_slide_ is the number of monomers between cross-links at the crack tip in the stretched state (Figure 11c). *N*′_slide_ is related to the sliding distance of the cross-linking points along the axis chains under stretching. As the slidable distance of the cross-links increases, *N*′_slide_ increases, and the toughness of the SR gels increases. *N*′_slide_ can be estimated from the measured fracture energy, *Γ*_SR_, using Equation (8). In the case of SR gels with 25% ring coverage, *N*′_slide_ is around 110–130, which is 16–18% of the entire axial chain [23]. As shown in Figure 10, the fracture energy of SR gels is independent of the Young’s modulus, suggesting that the slidable distance of the cross-links does not depend on the cross-linking density. This means that slide-ring cross-linking overcomes the trade-off relationship between the stiffness (Young’s modulus) and toughness (fracture energy) of polymer networks.

**Figure 12:**
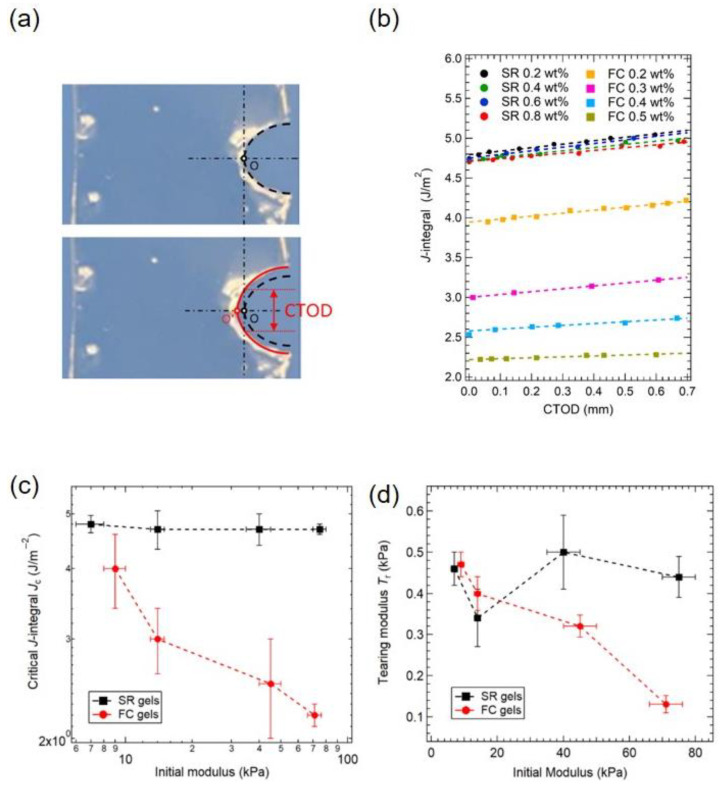
(**a**) Schematics for measuring crack tip opening displacement (CTOD), (**b**) J-integral—CTOD relationship of SR and FC gels with various cross-linker concentrations, (**c**) The initial modulus dependence of (**c**) critical J-integral *J*_c_ and (**d**) tearing modulus *T*_r_ of SR and FC gels. Reprinted from [30] with permission from Elsevier.

Furthermore, the crack propagation resistance of the SR gels was investigated by the J-integral crack tip opening displacement (CTOD) method [30]. J-integral is the energetic integral along a closed path around the crack tip, and CTOD is defined as the vertical intercept of the crack profile using the initial crack tip as the origin of the coordinate (Figure 12a). Figure 12b shows the J-integral—CTOD relationship for SR and FC gels with various cross-linker concentrations. For the FC gels, the J-integral values decrease with cross-linker concentration, while those of the SR gels are independent of cross-linker concentration. The value of J-integral at CTOD = 0.1 mm was defined as critical J-integral *J*_c_ representing the crack initiation resistance, and the slope of J-integral against CTOD was regarded as the tearing modulus *T*_r_ corresponding to the crack propagation resistance of the material. The values of *J*_c_ and *T*_r_ for the FC and SR gels are plotted against their initial (Young’s) moduli in Figure 12c,d. *J*_c_, which is equivalent to the fracture energy *Γ*, shows the same Young’s modulus dependence as *Γ*: *J*_c_ of the FC gels decreases with modulus whilst that of the SR gels is constant irrespective of initial modulus. The tearing modulus *T*_r_ also shows similar modulus dependence. For the SR gels, the crack propagation resistance remains constant regardless of the cross-linking density (Young’s modulus).

**Figure 13:**
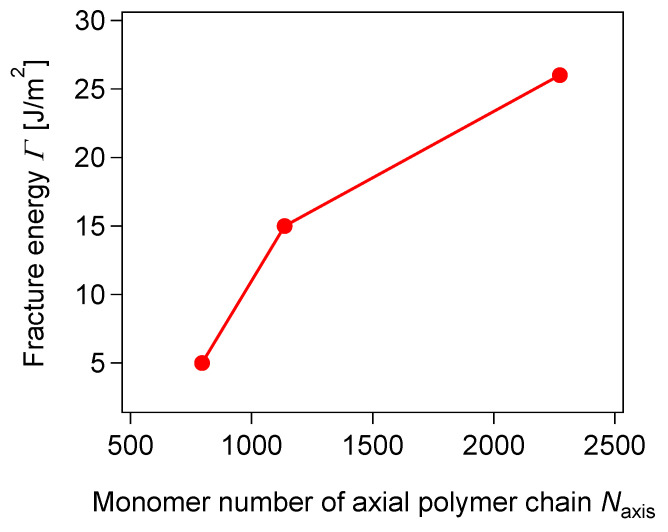
Fracture energy of SR gels with different molecular weights of PEG chains. Reprinted from [23] with permission from American Chemical Society.

**Figure 14:**
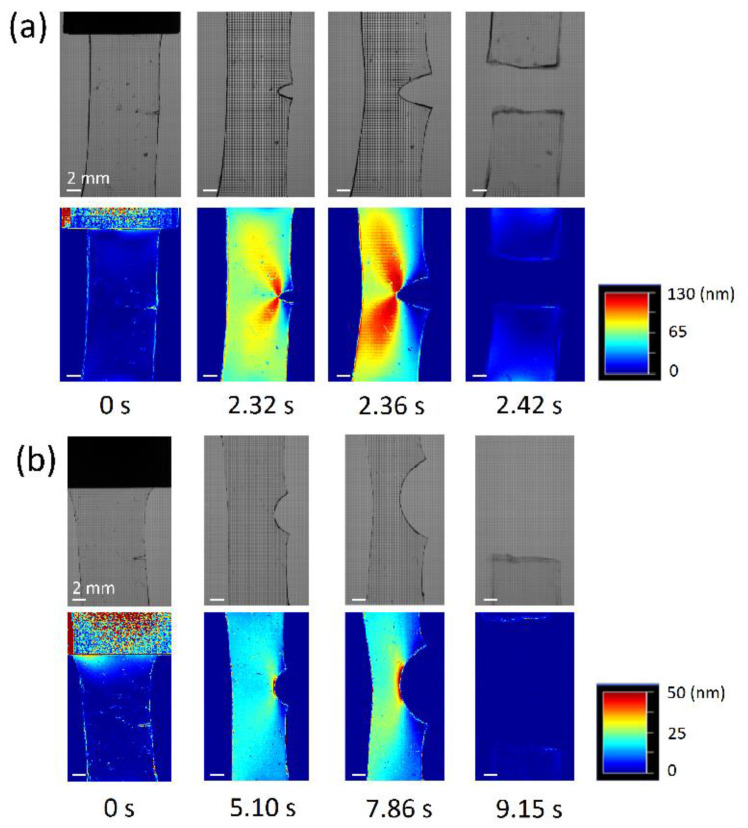
Gray scale photos and retardation maps of (**a**) SR gel with 25% CD coverage and (**b**) SR gel with 2% CD coverage during crack propagation. Reprinted from [29] with permission from IOP Science.

In order to enhance the toughness of SR gels, the slidable distance of the cross-links is enlarged by increasing the PEG chain length [23] and decreasing the CD coverage on PEG chains [28]. Figure 13 shows the fracture energies of SR gels with different PEG chain lengths (CD coverage: 25%, PR concentration: 10 wt%). The fracture energy and slidable distance *N*′_slide_ increase with the monomer number of PEG *N*_axis_ [23]. Jiang et al. successfully prepared a SR gel with only 2% CD coverage, and the fracture energy of the SR gel (PR concentration: 16 wt%) was approximately 50 J/m^2^ (*N*′_slide_ = 450, *N*′_slide_/*N*_axis_ = 65%), which is five-times larger than that of the SR gels with the CD coverage of 25%. Figure 14 shows the grey scale and retardation images captured by a polarized camera during the crack propagation process of the SR gels with 25% and 2% CD coverages, SR–25 and SR–02. For SR-25, the crack propagates through the specimen within 0.1 s, suggesting a catastrophic fracture. In contrast, the crack propagation in SR-02 gel is 40 times slower than that in SR-25 gel. The crack in SR-02 is blunted prior to the crack growth, and the crack blunting is related to the high crack resistance of SR-02.

## 5. Dynamic Fracture Behaviors of SR Gels

**Figure 15:**
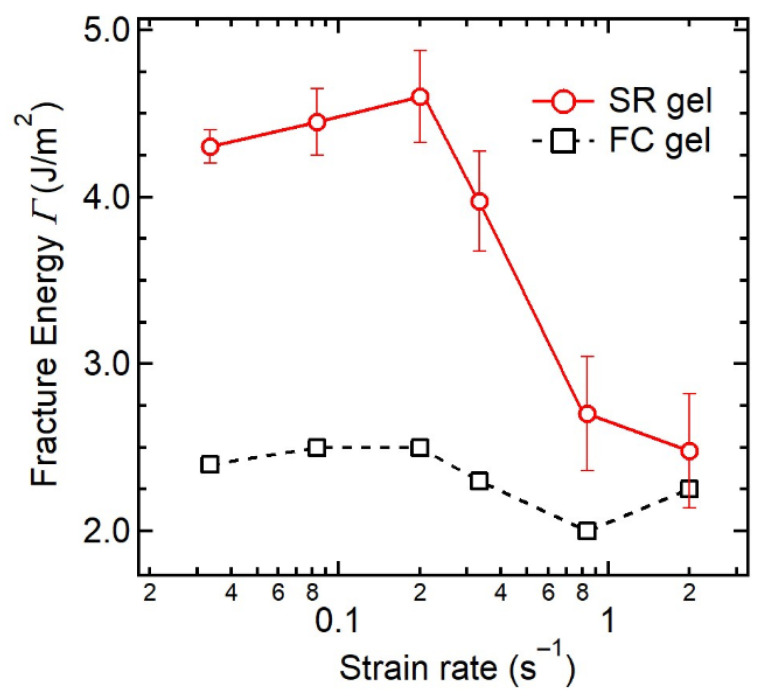
Strain rate dependence of fracture energy for FC and SR gels. Reprinted from [31] with permission from Elsevier.

**Figure 16:**
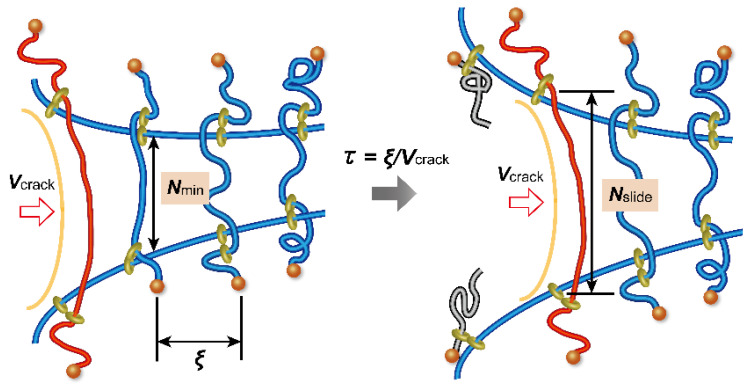
Schematic illustration of crack propagation model for SR gels. Reprinted from [31] with permission from Elsevier.

**Figure 17:**
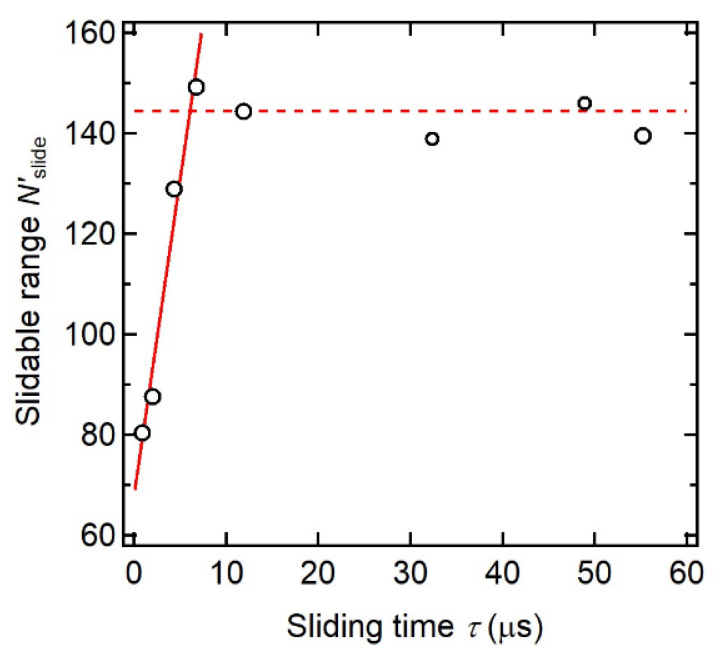
Plot of slidable range *N*_slide_ of slide-ring cross-links against sliding time *τ*. Reprinted from [31] with permission from Elsevier.

The dynamic fracture behavior of SR gels is correlated with the sliding dynamics of the cross-linking points. Liu et al. investigated the crack propagation behaviors of SR and FC gels in notched pure shear specimens under uniaxial stretching at various strain rates [31]. As shown in Figure 15, the fracture energy *Γ* of the FC gel is independent of strain rate, whilst that of SR gels decreases dramatically around at 0.3 s^−1^, indicating a transition of crack resistance in response to strain rate. At slow strain rates, the cross-links have enough time to slide along the polymer axes, which results in the higher toughness of the SR gel. For fast fracture, however, the sliding movement of the cross-links cannot catch up with the network deformation, and the cross-links behave as fixed cross-links. Figure 16 shows a simple molecular model for the dynamic fracture of SR gels. When a crack propagates at a velocity of *V*_crack_ from the first strand to the second one in the SR network with a mesh size *ξ*, the cross-links on the second strand slide along the chain during the time *τ* = *ξ*/*V*_crack_, which increases the monomer number of the second strand from *N*_min_ to *N*′_slide_. In Figure 17, *N*′_slide_ calculated from *Γ* by Equation (8) is plotted against the sliding time *τ*. The sliding range *N*′_slide_ increases with *τ* and is saturated at *τ* = 6 μs. This suggests that the cross-linking points in SR gels slide in micro seconds.

**Figure 18:**
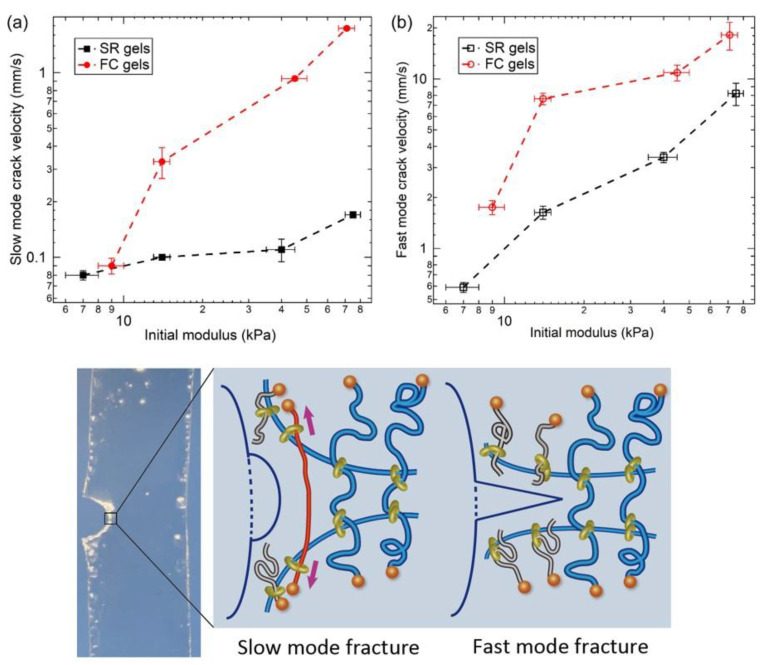
The initial modulus dependence of (**a**) slow mode crack velocity and (**b**) fast mode crack velocity for FC and SR gels. Reprinted from [30] with permission from Elsevier.

For single-edge notched tensile tests, the FC and SR gels show a slow-to-fast transition of crack propagation (Figure 18) [30]. During the slow mode fracture, the slide-ring cross-links have enough time to slide along the chains. Therefore, the slow mode crack propagation velocities of the SR gels are smaller than those of the FC gels, and independent of the Young’s moduli (Figure 18a). For the fast mode fracture, however, the deformation at the crack tip was faster than the sliding motion of the cross-links and the cross-links behaved as fixed ones, which results in the same Young’s modulus dependence of the fast mode crack propagation for the SR and FC gels (Figure 18b).

## 6. Dynamic Mechanical Response of SR Gels

**Figure 19:**
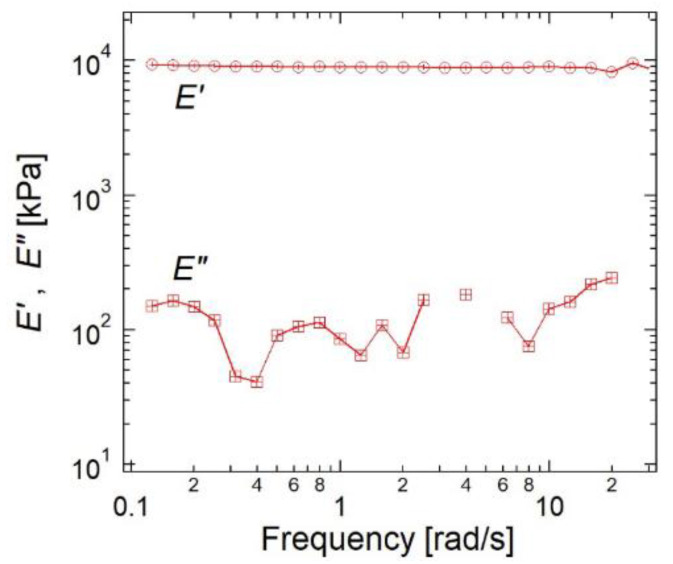
Frequency-dependence of *E*′ and *E*″ for SR gel. Reprinted from [23] with permission from American Chemical Society.

**Figure 20:**
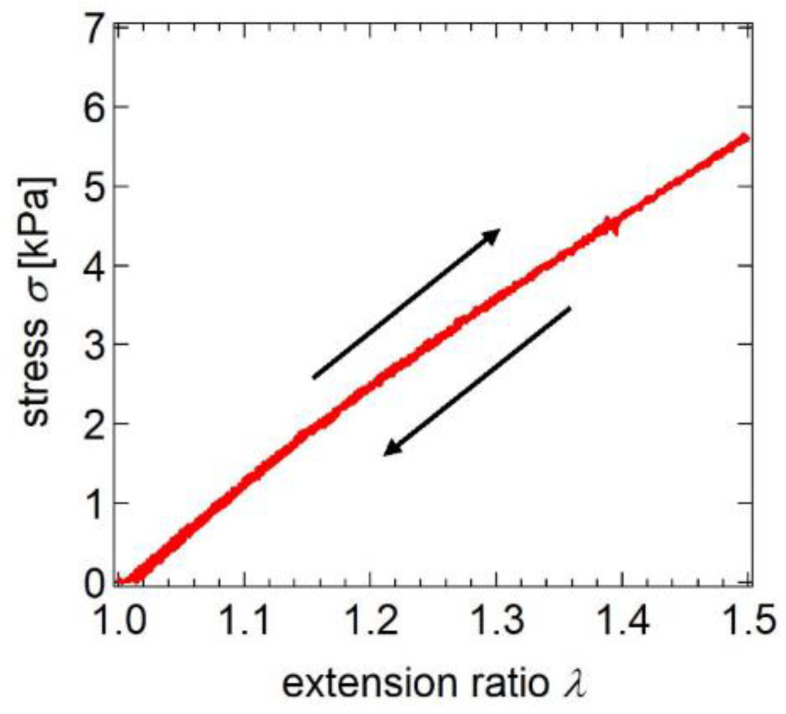
Stress–extension ratio curve of a loading–unloading cycle for SR gel. Reprinted from [23] with permission from American Chemical Society.

Although the sliding of the cross-links change the network structure of SR gels in response to strain, SR gels show elastic mechanical responses just as FC gels with fixed cross-links [20,23]. Figure 19 shows the frequency-dependence of the elastic modulus, *E*′, and loss modulus, *E*″, of the SR gel obtained from the linear viscoelasticity measurements at room temperature [23]. The SR gel exhibits a typical elastic mechanical response: *E*′ is independent of frequency and much larger than *E*″. In addition, SR gels are elastic under large deformation. As shown in Figure 20, there is almost no mechanical hysteresis during the loading–unloading cycles [23]. The observed elastic mechanical responses of the SR gels suggest that the mechanical relaxation resulting from the sliding of the cross-links may occur in a higher frequency regime (shorter time scale) than the experimental range, and that the sliding of the cross-links is much faster than macroscopic deformation, which is consistent with the microsecond-order sliding dynamics of the cross-links observed in the dynamic fracture behaviors (Section 5).

## 7. Solvent-Free SR Elastomers

**Figure 21:**
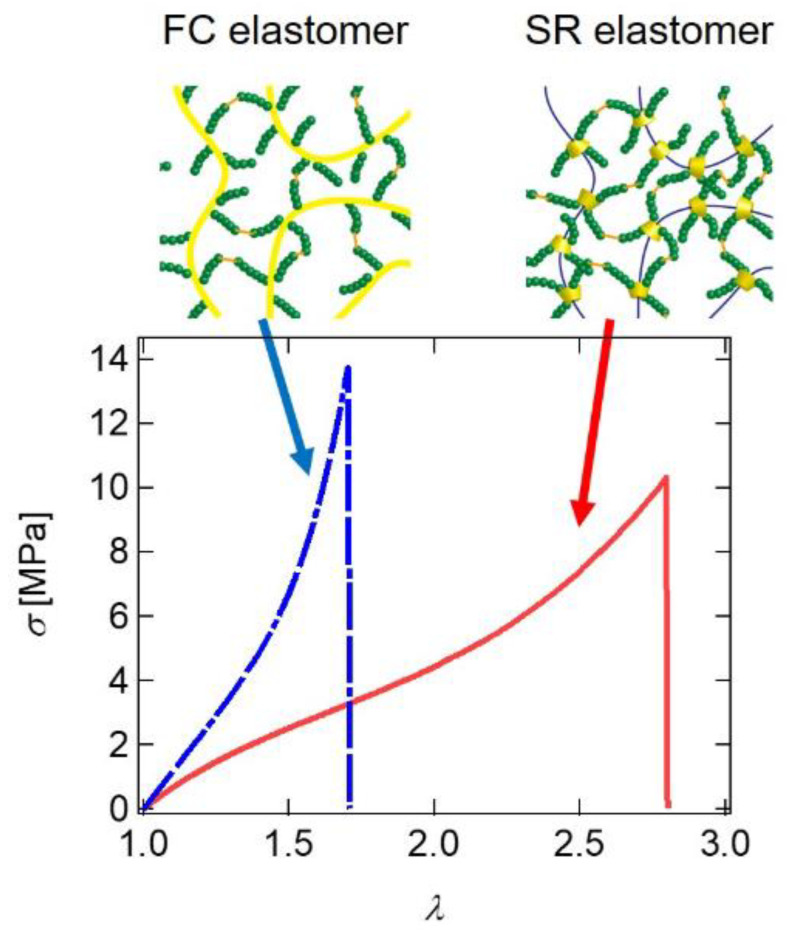
Stress–extension ratio curves of FC and SR elastomers. Reprinted from [36] with permission from Elsevier.

Slide-ring cross-linking is a general concept for network polymers that has been applied to solvent-free polymeric elastomers [35,36,37,38,39,40,41]. Araki et al. successfully fabricated solvent-free SR elastomers by cross-linking poly-ε-caprolactone (PCL)-grafted polyrotaxanes, in which PCL-modified CDs are threaded on PEG chains (Figure 21) [35]. The PCL graft chains were end-cross-linked via bifunctional cross-linkers. As a control, Minato et al. prepared FC elastomers from PCL–grafted hydroxypropyl cellulose and compared the mechanical properties between the FC and SR elastomers [36]. Figure 21 shows stress–extension ratio curves of the FC and SR elastomers with the same cross-linker concentration. The SR elastomer is softer and shows a higher strain at rupture than the FC elastomer, which is similar to the SR gels. The softness and high extensibility of the SR elastomer originates from the slidability of the cross-links. The SR elastomer has been used for scratch-resistant coatings and dielectric elastomer actuators [39].

**Figure 22:**
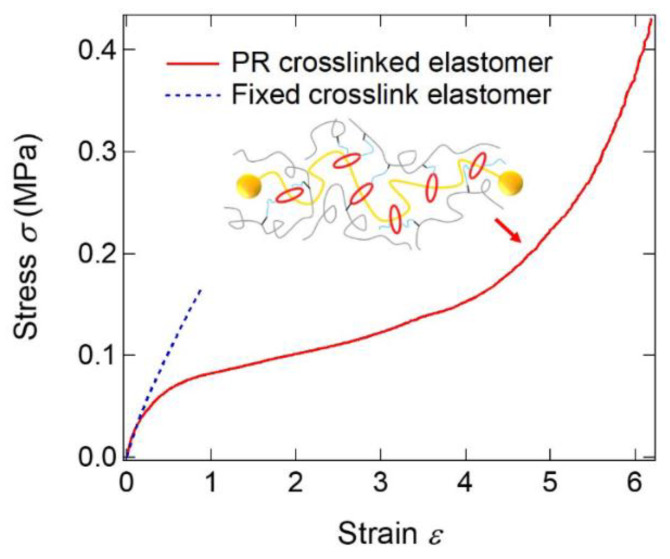
Stress–strain curves of fixed cross-link and PR cross-linked elastomers. Reprinted from [40].

Goto et al. introduced slide-ring cross-linking points into polymer networks by polymerizing monomers with PR cross-linkers containing vinyl groups on CDs [40]. Only 1% addition of PR cross-linker improved significantly extensibility and toughness compared with FC elastomers (Figure 22). The PR cross-linked elastomer shows a streak in SAXS patterns under stretching. This indicates that the PR cross-linkers are highly stretched and oriented in the elastomer, which leads to stress dispersion by the network transformation of the SR network. Sawada et al. reported that crown ether-based rotaxane cross-linkers are also useful to obtain tough elastomers [41].

**Figure 23:**
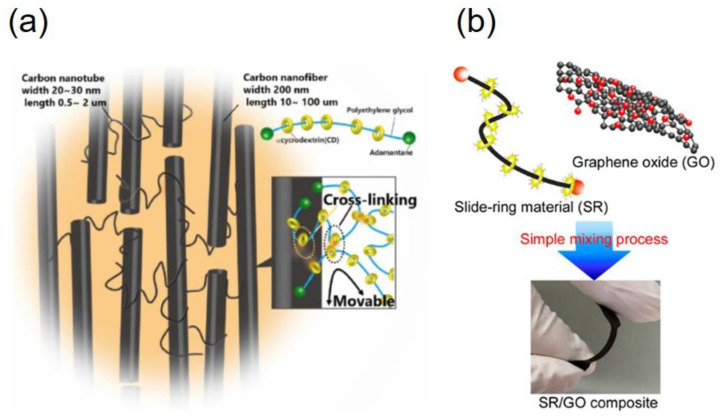
Schematic illustration of (**a**) SR elastomers with plasma-surface-modified carbon nanofiber (CNF)/carbon nano-tube (CNT) [43] and (**b**) SR graphene oxide (GO) composites with high electrical conductivity and mechanical toughness [44]. Reprinted from [44] and [45] with permission from Elsevier and American Chemical Society, respectively.

Recently, SR elastomers have been used for composite materials [42,43,44,45]. Goto et al. developed tough, flexible, and highly thermally conductive SR elastomers with plasma-surface-modified carbon nanofiber (CNF)/carbon nanotube (CNT) (Figure 23a) [43]. Although the SR composite contains 50 wt% of CNF/CNT, the toughness of the SR composite is higher than raw SR elastomer without fillers, and the Young’s modulus is still in the elastomer regime. Hatakeyama et al. reported SR graphene oxide (GO) composites with high electrical conductivity and mechanical toughness and used the SR composite as stretchable base substrates for a humidity sensor, motion sensor, and electrical heater (Figure 23b) [44]. Howell et al. fabricated a stretchable one-dimensional photonic crystal using SR elastomers containing zirconium dioxide particles [45]. The SR nanocomposite with 70 wt% zirconium dioxide nanoparticles have a high refractive index without losing mechanical softness and elasticity, which enables the strain-tunable photonic crystal with high refractive index contrast.

## 8. Conclusions and Future Perspectives

In this article, we reviewed the mechanical/fracture properties of SR gels with movable cross-links and discussed the molecular origins of their softness, elasticity, and toughness. When SR gels are stretched, the sliding of the cross-links weakens chain deformation in the stretching direction, which reduces the Young’s modulus. The low Young’s modulus of SR gels is formulated by a simple equation as a function of *N*_slide_, that is, the degree of sliding of the cross-links. The sliding of the cross-linking points also enlarges the partial strand length between cross-links at the crack tip, which leads to high toughness of SR gels. The fracture energy of SR gels is dominated by the sliding distance of the movable cross-links, represented by *N*′_slide_. Reducing the ring coverage on the polymer chains and/or increasing the axial polymer chain length leads to the increase of *N*′_slide_, and enhancement of the mechanical toughness. Although the network structure of SR gels is transformed under deformation, SR gels show elastic mechanical responses (no hysteresis loss) over a wide range of strains, suggesting that the sliding dynamics of the cross-links is much faster than macroscopic deformation. For the crack propagation tests, the stretching velocity at the crack tip is much higher than the global deformation rate; therefore, we successfully evaluated the sliding dynamics of the cross-links from the dynamic crack propagation behaviors. The estimated time scale of the sliding motion was of the microsecond-order, which is much faster than global mechanical deformation. The concept of the SR network is applicable not only to gels, but also to non-solvent elastomers and composite materials to improve mechanical toughness. For a future perspective, investigation of the slide-ring effect in polymeric resins would be an important and interesting direction in this field.
